# Artificial Intelligence for Diagnosis of Esophageal Manometry: A Narrative Review

**DOI:** 10.1007/s11894-026-01052-3

**Published:** 2026-08-10

**Authors:** Ernesto Robalino Gonzaga, Juliana J. Madej, Nitin Ahuja

**Affiliations:** 1 Division of Gastroenterology and Hepatology, Department of Medicine, Perelman School of Medicine, University of Pennsylvania, 3400 Civic Drive Boulevard, Philadelphia, PA 19104 USA; 2 Idaho College of Osteopathic Medicine, 1401 E Central Drive, Meridian, ID 83642 USA

**Keywords:** Artificial intelligence, High resolution manometry, Esophageal motility disorders, Deep learning, Chicago classification, Medical education

## Abstract

**Purpose of Review:**

High-resolution manometry (HRM), the gold standard for diagnosing esophageal motility disorders, remains constrained by inter-rater variability, limited access to expert esophagologists, and inadequate training infrastructure. Many gastroenterology fellowship programs provide insufficient motility training, and dedicated expert centers remain geographically concentrated. Emerging Artificial intelligence (AI) tools have the potential to automate diagnosis, and augment clinician interpretive capacity and democratizing expert-level motility assessment.

**Recent Findings:**

Twenty-two studies encompassing over 5,000 patients were synthesized across three overlapping developmental phases: early machine learning for feature extraction and classification; deep learning deployed toward automated pattern recognition and motility classification and emerging multimodal and large language model–based frameworks augmenting clinical interpretation and decision-making, with diagnostic accuracies ranging from 71 to 97%. AI integration of multimodal manometric data may reveal pressure signatures imperceptible to human visual inspection, pointing toward phenotypes beyond the Chicago Classification. No study has yet demonstrated improved patient outcomes. AI’s role in motility training remains largely unexplored.

**Summary:**

The AI–clinician partnership represents the most promising trajectory for esophageal manometry interpretation in coming years. AI-augmented interpretation has the potential to eliminate the two-tier diagnostic gap between academic and community practice, to accelerate trainee competency through scalable AI-supervised case libraries, and to uncover physiologic phenotypes beyond human interpretation.

**Supplementary Information:**

The online version contains supplementary material available at 10.1007/s11894-026-01052-3.

## Introduction

Esophageal motility disorders affect a significant proportion of patients presenting with dysphagia, chest pain, refractory reflux. These include disorders of the esophagogastric junction (EGJ) such as achalasia types I through III and EGJ outflow obstruction (EGJOO); and disorders of peristalsis such as absent contractility, ineffective esophageal motility (IEM), distal esophageal spasm (DES), and hypercontractile esophagus [1]. 

High-resolution manometry (HRM) has become the cornerstone diagnostic tool for esophageal motility disorders. HRM generates detailed spatiotemporal pressure topography recordings that surpass the capabilities of conventional line tracing. The Chicago Classification (CC), now in its fourth iteration (CCv4.0), provides a structured algorithmic framework for translating HRM pressure data into clinical diagnosis, incorporating upright and supine positions, provocative maneuvers, and refined esophagogastric junction (EGJ) metrics [1]. 

That said, HRM interpretation is far from straightforward. The sheer volume of data obtained through a standard manometry protocol presents a practical challenge. Each study employs catheters with 36 channels and 432 discrete points of measurement per catheter assembly, generating a spatiotemporal pressure dataset of considerable complexity [2]. This interpretive burden demands time and expertise, resources that are not uniformly available across practice settings.

The American Neurogastroenterology and Motility Society (ANMS) registry lists 107 dedicated GI motility laboratories across the United States as of 2025 [3], a substantial increase from the 38 centers documented a decade earlier [4]. These laboratories remain concentrated in academic medical centers and major urban areas as opposed to rural and community settings. Establishing a motility laboratory demands considerable infrastructural investment and requisite referral volume. The hiring and training of laboratory-affiliated staff (from procedural operators to billing/coding specialists) and acquisition and maintenance of the requisite equipment may be cost-prohibitive outside high-volume centers [5]. 

The complexity of CCv4.0 demands substantial training and expertise. Even among trained experts, diagnostic agreement is imperfect. A 2018 analysis by Kim and colleagues demonstrated that inter-rater agreement for HRM-based motility diagnosis achieves only fair concordance (Kappa 0.40) [6]. The subjective element of HRM interpretation introduces meaningful diagnostic uncertainty even at experienced centers. For gastroenterologists without dedicated motility training and/or based in relatively low-volume community settings, this variability is likely even greater.

The scope of this problem is accentuated by a well-documented motility training gap. A survey of 171 adult gastroenterology fellowship programs in the United States found that only 25% provide dedicated training in gastroenterology motility [4]. This was later corroborated in a study of pediatric gastroenterology fellows’ experience, with 75% of surveyed trainees reporting inadequate training in NGM during fellowship, 80% of whom received two weeks or less of dedicated motility exposure [7]. Dedicated Neurogastroenterology and Motility fellowship programs have grown from fewer than 10 in 2015 to approximately 13–15 currently active programs across the United States, collectively training an estimated 15–20 fellows annually, a pipeline insufficient to meet growing clinical demands [5]. 

The diagnostic opportunity latent in contemporary HRM data depends on the existence of tools to interpret that consistently, reproducibly, and at scale. Artificial intelligence (AI) offers these capabilities. AI encompasses machine learning (ML) and deep learning (DL), utilizing specific architectures such as convolutional neural networks (CNN) and transformer-based large language models (LLM). In gastroenterology, AI is already transforming upper endoscopy and colonoscopy through real-time polyp detection and lesion characterization. While applying AI to other physiologic testing modalities has lagged [2], this application holds the potential not only to automate manometry interpretation but also to address the structural access problem by decoupling diagnostic quality from geographically concentrated expertise, and by serving as a novel pedagogical tool that accelerates competency acquisition in a field where training is scarce.

This narrative review synthesizes the current evidence for AI as applied to esophageal manometry, with a focus on diagnostic accuracy, treatment prediction, methodological quality, and practical requirements prior to responsible clinical implementation. A central theme throughout this review is the framing of AI as a clinical collaborator; that is to say, AI can augment the gastroenterologist’s interpretive reach and amplify the motility expert’s training capacity, rather than serving as an autonomous technology operating independently of human judgment. This human-AI partnership model has direct implications for how the field should develop training curricula, design validation studies, and approach the structural access problem in motility care [2, 8, 9]. 

## Methods

**Fig. 1:**
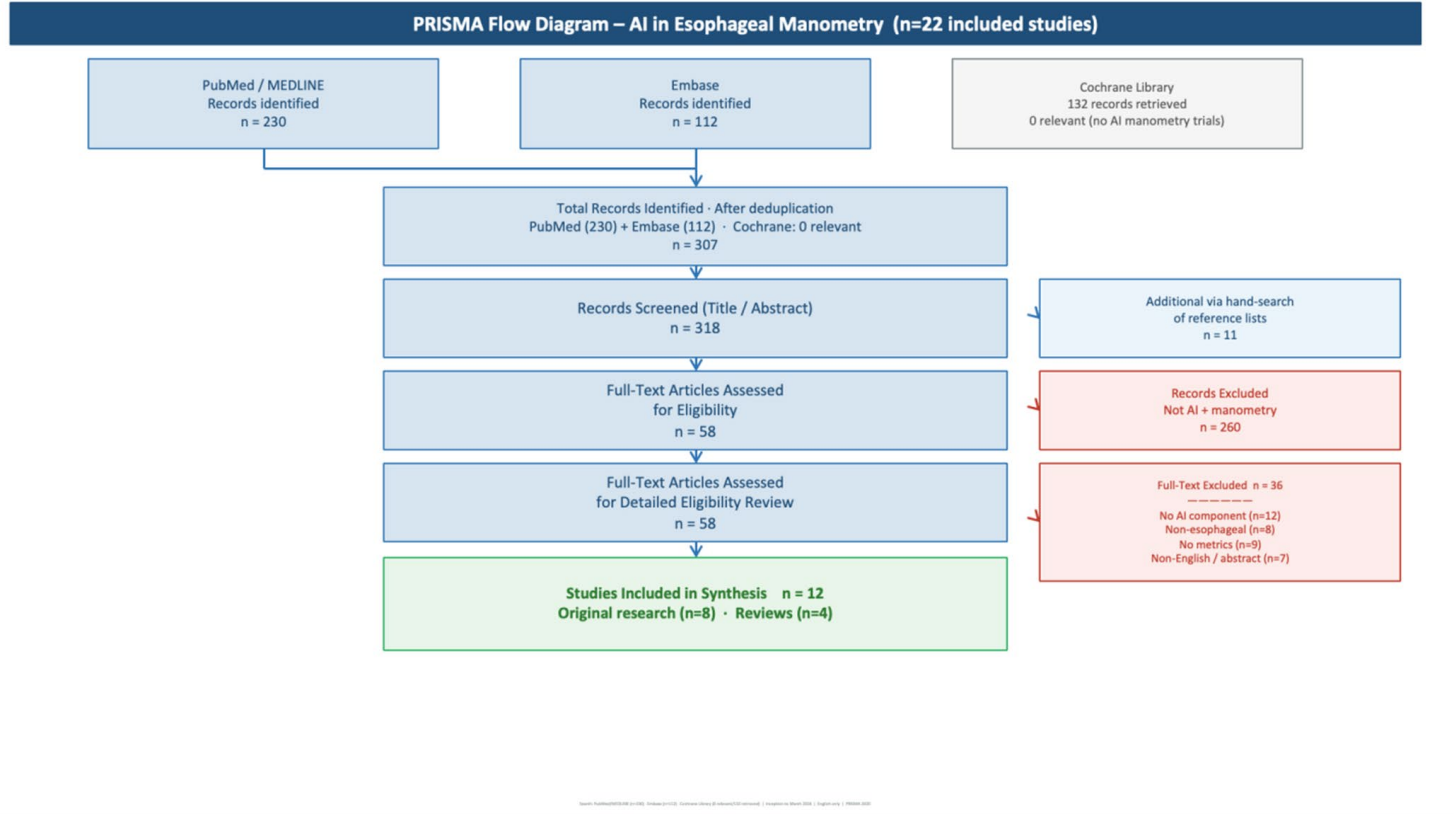
PRISMA 2020 flow diagram of study identification, screening, and inclusion. Records were identified through searches of PubMed/MEDLINE, Embase, and the Cochrane Library (inception through March 2026), supplemented by hand-searching reference lists of included reviews. After deduplication and addition of hand searched records, 318 records were screened by title/abstract; 260 were excluded as not addressing AI applied to manometry. Full-text articles were assessed against the pre-specified inclusion/exclusion criteria in Table 1, with reasons recorded for each exclusion. Twelve studies (8 original research, 4 reviews) met inclusion criteria and were included in the qualitative narrative synthesis. AI = artificial intelligence

This narrative review was conducted via a structured, PRISMA-informed literature search. Its evidence base was identified through searches of PubMed/MEDLINE, Embase, and the Cochrane Library from inception through March 2026 (Fig. 1). Given the heterogeneity of extant study methodologies, populations, and outcome measures in this field, our findings are presented as a qualitative narrative synthesis. Inclusion and exclusion criteria are summarized in Table 1. Studies were included if they applied AI, ML, or DL to interpret esophageal motility data from any modality in adult patients and reported extractable diagnostic performance metrics. Case reports, editorials, and conference abstracts without full-text data were excluded.

**Table 1:** Inclusion and exclusion criteria

Criterion	Inclusion	Exclusion
Population	Adults with esophageal symptoms undergoing manometry or FLIP	Pediatric only studies; animal studies
Intervention	Any AI, ML, or DL method applied to esophageal motility data	Manual interpretation studies without AI component
Comparator	Expert human interpretation, Chicago classification, or no comparator	
Outcome	Diagnostic accuracy, motility classifications, pattern recognition, or outcome prediction	Studies without extractable performance metrics
Study Type	Original research, systematic reviews, validation studies, methodological studies	Case reports; editorials; conference abstracts
Modality	All esophageal manometry modalities (HRM, FLIP, HRIM, acoustic)	Exclusively anorectal or pharyngeal manometry studies
Language	English	Non-english language publications
Time Frame	All years to March 2026	

Abbreviations: *AI* Artificial intelligence, *ML* Machine learning, *DL* Deep learning, *HRM *high resolution manometry

Specific search terms are provided in the supplementary information.

Results were limited to English-language publications at the screening stage. PubMed/MEDLINE yielded 230 records, and Embase 112, yielding a total of 342 records before duplicates were removed. The Cochrane Library was additionally searched but returned no relevant records. Reference lists of included reviews were hand-searched to identify additional studies (11 additional records identified) with included studies summarized in Table 2. The systematic search was designed exclusively to identify studies applying AI or ML to esophageal motility data. References cited for contextual framing of the access and training infrastructure gap [4, 7, 18] were identified via a separate targeted narrative search not subject to the PRISMA screening process. Seven conference abstracts were identified, excluded per pre-specified criteria (absence of full-text peer-reviewed publication) but cited narratively as emerging evidence.

**Table 2:** Included study characteristics

Ref.	Author, Year	AI Method	CC Version	*n*	Task	Performance	Key Findings
[10]	Kou, 2022	CNN + ANN + XGBoost (multi-stage)	CCv3.0	1,741 HRM studies	Swallow-type & pressurization classification; IRP estimation; study-level diagnosis	Swallow-type accuracy 0.88; pressurization accuracy 0.93; IRP MAE 4.5 mmHg; top-1 precision accuracy 0.81; top-2 precision accuracy 0.92	Landmark multi-stage model mirroring CC hierarchical logic; weighted IRP loss function; rule-based ceiling 76.6%
[11]	Kou, 2021	Variational Autoencoder (VAE); unsupervised DL	CCv3.0	32,415 swallows; 2,161 studies	Unsupervised latent clustering of raw HRM pressure data (no diagnostic labels)	6-class swallow accuracy 0.64/0.63/0.64 (train/val/test); 3-class pressurization accuracy 0.87/0.86/0.87	First unsupervised DL on raw HRM data; emergent clusters recapitulated CC categories; revealed continuous diagnostic boundaries
[12]	Popa, 2024	CNN + Gemini LLM (multimodal)	CCv3.0	926 HREM images	Automated HREM image classification; LLM-assisted contextual interpretation	Precision 0.89; Accuracy 0.88; F1 0.885	LLM-augmented multimodal framework; contextualizes manometric findings with clinical data
[13]	Czako, 2021	CNN + supervised ML	Not specified	ImageNet pretrain; HRM test set	IRP classification; probe mispositioning detection (quality control)	IRP accuracy 97%; normal IRP F1 96%; elevated IRP F1 98%; probe positioning accuracy 91%; mispositioning F1 93%	First AI quality-control application; addresses probe mispositioning as recognized source of diagnostic error
[14]	Kou, 2022	LSTM deep learning	CCv3.0	26,115 swallows; 1,741 studies	Swallow-type identification; study-level peristalsis classification	Swallow-level accuracy 0.83; study-level peristalsis accuracy 0.88	LSTM captures temporal swallow dynamics; feeds into multi-stage study-level pipeline
[15]	Wang, 2021	Conv3D + BiConvLSTM (EMD-DL)	Not specified	229 patients	Esophageal motility function classification over time	Accuracy 91.32%; sensitivity 90.5%; specificity 95.87%	Temporal DL in independent Chinese cohort; adds geographic diversity to North American-dominated literature
[16]	Surdea-Blaga, 2022	InceptionV3 + DenseNet201 (two-stage DL)	CCv3.0	192 patients; 1,535 images	Fully automated CC diagnosis from HRM Clouse plot images	Accuracy and F1 score of 86%	Two-stage image-based pipeline; Romanian cohort (ISOLAB, IRP ULN 28 mmHg); raises reference standard comparability concern
[17]	Geiger, 2025	DL swallow detection + unsupervised clustering	CCv4.0	25 LTHRMs; >23,000 images	Automated swallow detection and clustering for 24-hour ambulatory HRM	Detection precision 86.1%; recall 94.1%; F1 89.6%	First AI enabling clinical feasibility of LTHRM; reduces interpretive burden to representative swallow set

## AI Taxonomy and Terminology

A working understanding of AI terminology is a prerequisite for interpreting the manometry literature. ML refers broadly to algorithms that learn patterns from labelled data without explicit programming. Traditional ML approaches including random forests, support vector machines (SVM), and gradient boosting. ML extracts pre-defined features such as integrated relaxation pressure (IRP) or distal contractile integral (DCI) and uses them to classify studies. These methods, while computationally efficient and relatively interpretable, depend heavily on the quality and completeness of feature engineering.

DL employs multi-layered artificial neural networks that learn feature representations, raw data converted into numerical vectors that AI algorithms can process and analyze. CNNs, the dominant DL architecture in image-based manometry research, are particularly suited to spatiotemporal pressure topography plots [10]. Unsupervised DL approaches, such as variational autoencoders (VAEs), learn latent data representations without labelled training examples, which offers the potential to discover novel motility phenotypes not captured by current classification schemes [11]. LLMs and multimodal generative AI represent the most recent frontier within the DL paradigm, capable of processing both image and text data simultaneously [12]. 

Interpreting AI diagnostic performance requires familiarity with a small set of statistical metrics that differ meaningfully from the sensitivity and specificity familiar to clinicians. These derive from the confusion matrix, also called an error matrix, which represents a simple tabulation of true positives (TP), true negatives (TN), false positives (FP), and false negatives (FN) for a given diagnostic category. Accuracy (TP + TN / total) is the most reported metric in the manometry AI literature but is also the most misleading in imbalanced datasets. As an example, a model that correctly identifies all normal studies while misclassifying every achalasia case may still yield > 90% accuracy if the normal class dominates the dataset.

Precision (TP / TP + FP) reflects how often a positive prediction is correct, analogous to positive predictive value. Recall (TP / TP + FN) reflects how many true positives are captured, analogous to sensitivity. The F1-score is their harmonic mean, providing a single balanced metric particularly useful when class distributions are uneven. AUROC (area under the receiver operating characteristic curve) measures discrimination across all possible decision thresholds, independent of prevalence. The distinctions among these metrics matter clinically. A system with 95% overall accuracy but poor recall for type III achalasia, for example, may systematically miss the patients most likely to benefit from Peroral Endoscopic Myotomy (POEM).

## Artificial Intelligence for Diagnosis in High Resolution Manometry

### Landmark Identification and Quality Control

The technical quality of an HRM study must be assured by anatomical landmark identification, particularly the EGJ and upper esophageal sphincter. Czako and colleagues addressed this foundational step using ML to simultaneously classify IRP and detect probe positioning failures, achieving an accuracy of 97% for IRP classification with a F1-score over 84% in both cases [13]. This application is clinically significant, as probe malpositioning is a recognized source of diagnostic error that current software handles inconsistently across platforms [13]. Automating its detection, particularly during real-time recording, could meaningfully reduce the volume of technically inadequate studies.

### Swallow-Level Classification

The Chicago Classification operates through a hierarchical, stepwise algorithm that first characterizes individual swallows before deriving a study-level diagnosis. Kou and colleagues developed a DL long short-term memory (LSTM) model, an enhanced subtype of recurrent neural networks that function by utilizing internal gating mechanisms to control the retention and flow of information across sequential data, for swallow type identification [14]. This achieved 83% test accuracy for swallow-level classification and 88% for study-level peristalsis classification [14]. Wang and colleagues applied three-dimensional convolution (Conv3D) and bidirectional convolutional LSTM (BiConvLSTM) networks to capture temporal dynamics of motility in an independent Chinese cohort, achieving 91.3% accuracy [15]. The latter is noteworthy as one of the few studies demonstrating generalizability beyond the North American centers that have dominated this literature. Notably, two conference abstracts, Kern and colleagues (DDW 2018) applying ML to HRM isocontours and Huang and colleagues (DDW 2019) reporting a deep learning proof-of-concept, predate all full-text publications in this area.

### Study-Level Diagnosis and Chicago Classification Automation

The methodological landmark in HRM AI is the multi-stage framework developed by Kou and colleagues at Northwestern [10]. The model mirrors the CC algorithm architecture: DL-based CNNs operate at the swallow level, followed by feature-based ML models (XGBoost and artificial neural networks) at the study level. This hierarchical design embeds knowledge about how expert clinicians reason through manometry data. Notably, the IRP sub-model employs a weighted loss function that applies greater training penalties for prediction errors near the 15mmHg diagnostic threshold, penalizing clinically consequential misclassifications more heavily than errors in unambiguous ranges. Systematically tuning the IRP cutoff and proportion thresholds produced almost no accuracy gain (76.6% vs. 76.2% for standard values). This showed that the problem was not which threshold values were used, but the rule-based decision-tree method itself, which had a built-in performance limit that no amount of threshold-tuning could overcome. The authors therefore replaced this rule-based approach with more flexible machine-learning models, including convolutional neural networks and gradient-boosted trees, which raised study-level diagnostic accuracy to nearly 90% [10]. 

A complementary unsupervised approach using a variational autoencoder (VAE), which compresses raw HRM swallow data into a probabilistic latent representation without requiring diagnostic labels, was trained on over 32,000 swallows from 2,161 HRM studies [11]. Unlike every other study cited in this manuscript, the VAE was never shown a CC diagnosis during training; it learned purely from pressure patterns. The emergent latent clusters broadly recapitulated CC diagnostic categories, simultaneously validating the biological basis of the classification and revealing that the boundaries between diagnostic categories are continuous rather than discrete. This has direct implications for our collective clinical understanding of borderline and overlap phenotypes.

Surdea-Blaga and colleagues independently developed a fully automated Chicago Classification system in a Romanian cohort of 192 HRM studies, achieving 86% accuracy and F1-score [16]. Methodologically, this is not a single model but a two-stage deep learning pipeline. InceptionV3 for IRP classification, followed by DenseNet201 for swallowing disorder categorization, have outputs that feed into a CC decision tree to generate the final diagnosis. This architecture is conceptually similar to the Kou multi-stage framework [10]. Both mirror the CC’s hierarchical logic by separating earlier physiological parameter estimation from final diagnostic classification. The key distinction is that Kou operates on raw time-series pressure data, while Surdea-Blaga operates on image representations of the Clouse plot, reflecting the two dominant paradigms in HRM AI – raw-data versus image-based classification. The Romanian cohort also introduces important geographic diversity to the literature. Given that the overwhelming majority of HRM AI development has occurred at North American centers using Medtronic hardware, this group used an ISOLAB system with a Unisensor catheter, which carries a different IRP upper limit of normal (28 mmHg vs. 15 mmHg), raising a methodological concern about reference standard comparability that the study does not explicitly address.

### Long-Term and Ambulatory High-Resolution Manometry

Geiger and colleagues addressed the analytical burden of 24-hour long-term high-resolution manometry (LTHRM) with a deep learning pipeline that detects more than 94% of all relevant swallow events and employs unsupervised clustering to organize detected swallows into distinct classes, allowing clinicians to focus on a small number of representative swallows in a manageable, clinically interpretable summary [17]. This approach makes LTHRM clinically feasible for the first time, with implications for evaluating intermittent dysphagia and post-surgical motility assessment across a full circadian cycle.

## Methodological Critique and Limitations of the Current Evidence Base

All AI systems reviewed in this manuscript are trained and validated against expert clinical diagnoses based on CCv4.0 classifications assigned by experienced esophagologists. However, Kim et al. demonstrated that this reference standard achieves only fair inter-rater concordance (κ = 0.40) [6]. As highlighted by Fass, Rogers, and Gyawali and likewise noted by Gong et al., existing AI systems compare AI interpretation to expert analysis rather than to clinical outcomes from management based on AI diagnosis [2, 9]. If expert diagnosis itself is imperfect as the inter-rater variability data clearly demonstrate [6], then training AI to match expert opinion merely encodes existing diagnostic uncertainty into a more consistent format. An AI model that faithfully replicates expert opinion does not provide better diagnoses; it provides more consistent versions of the same subjective judgements. This creates an epistemological ceiling that cannot be overcome unless AI training targets are linked to clinical outcomes rather than expert labels.

Calibration remains largely unaddressed, with almost no study reporting calibration metrics such as Brier scores or calibration curves. As a result, probability outputs cannot be assumed to reflect true clinical probabilities, and overconfident predictions in borderline cases remain an unquantified risk. Importantly, because the reference standard is based on expert opinion rather than objective biological truth, even a well-calibrated model would only predict agreement with experts’ current understanding rather than true diagnostic accuracy. A further concern is reference standard inconsistency. Studies span Chicago Classification versions 3.0 and 4.0,^5^ which differ substantially in diagnostic thresholds and category definitions.

Crucially, unsupervised approaches such as the variational autoencoder model of Kou and colleagues suggest a second complementary trajectory: AI trained on raw pressure data without diagnostic labels may identify latent manometric signatures invisible to human visual inspection, potentially revealing motility phenotypes that lie outside the boundaries of the current classification framework. In this sense, AI’s most disruptive contribution may not be automating what experts already see but surfacing what they cannot.

ML-specific concerns include sample size adequacy. CCv4.0 defines at least eight distinct motility phenotypes, yet most models are trained on datasets ranging from 192 to 1,741 studies with variable class distributions. These models are therefore at risk of overfitting to common diagnoses while underperforming on rare but clinically significant conditions such as type III achalasia, absent contractility, and hypercontractile esophagus. No study reports category-specific performance metrics, making this limitation difficult to quantify.

Methodological transparency is also limited. While some studies report stratified train-test splits, most do not specify how datasets were partitioned or whether class distributions were preserved. Combined with the absence of external validation and unclear patient selection processes, this raises concern for optimistic performance estimates, particularly for rare diagnoses. Along similar lines, a model achieving high accuracy on an internal test set derived from the same single-center dataset provides limited insight into real-world performance. Generalizability and replicability across institutions, patient populations, manometry systems, and protocol variations remains uncertain.

Largely invisible to systematic reviewers is the body of conference abstracts that have never progressed to full publication. Seven abstracts [19–22] collectively spanning seven years at major international meetings confirm that conference-stage AI activity substantially exceeds the published evidence base. Without full peer-reviewed publication, a form of reporting bias inflates the apparent quality and maturity of the field, while the MRS gap identified by Lei Wei-Yi et al. highlights a clinically important task that published AI has not yet addressed.

The evidence base is also affected by systematic selection bias toward high-volume expert centers. High-performing models, such as the Northwestern multi-stage framework [10] and the LTHRM framework [17], originate from specialized motility units with expert annotators and high procedural volumes. Given that only a minority of training programs offer formal motility training [18] and that relatively few dedicated motility centers exist across North America [3], most real-world users differ significantly from the populations represented in training data. This creates a paradox: AI trained on expert-center data may fail in community settings where it is most needed. External validation at non-specialist centers is therefore not merely a methodological formality, but rather the defining test of whether AI can fulfill its democratizing potential.

Finally, to our knowledge, no AI manometry tool has yet received regulatory clearance from the FDA or CE marking as a clinical diagnostic device. Algorithmic transparency, failure mode characterization, and integration into existing clinical workflows are prerequisites for responsible clinical translation.

## Future Directions and Expert Perspective

A central research and development core for AI in HRM could serve to address the current methodological inconsistencies present in the literature and establish a pipeline for accelerated development of AI models. This would be twofold, to create models both for clinical practice and for motility training. As a recent example from the realm of endoscopic pattern recognition, a Danish multicenter data set serving as a foundation model with anonymized generalized endoscopic images from multiple hospitals outperformed other AI models in accuracy, demonstrating the power of a centralized data core [23]. Similarly a centralized data set of existing and future manometry records serving as a case library could help with pretraining AI models, advancing AI innovation workflows, investigating model errors, and partnering with industry manufacturers to integrate AI with existing manometry tools. The esophageal manometry competency program, a personalized adaptive learning program for HRM interpretation, improved trainee performance across all seven core manometry skill sets, yet its reach is limited by the small number of institutions with expert faculty capable of supervising it [18]. Realizing this potential will require deliberate collaboration from professional societies, neurogastroenterologists, educators, and AI developers.

An AI-supervised training platform functions simultaneously as teacher, examiner, and quality-assurance mechanism. When built on an extensive case library, an AI training platform could provide instant feedback on trainees’ interpretation against the AI benchmark, adapt case complexity to individual performance trajectories, and identify knowledge gaps requiring targeted remediation without requiring physical access to a dedicated motility laboratory or expert mentor. That is to say, AI enables a distributed training architecture: trainees acquire interpretive competency with AI and then validate that competency in supervised clinical practice. This idealized model does not eliminate the role of expert motility physicians; it merely amplifies their reach. A single experienced neurogastroenterologist can curate and validate the annotated case library that trains hundreds of trainees across dozens of institutions.

Standardized AI-assisted training curricula aligned with the ANMS-ESNM tiered competency framework [4], prospective assessment of trainee outcomes under AI-supervised versus traditional training models, and regulatory frameworks that distinguish AI as a training tool from AI as a clinical diagnostic device are all necessary components of an optimized and sustainable future model. The field is at an inflection point: the AI tools exist, the training gap is documented, and the collective need is clear. What is required is the institutional will to build the bridge.

### Multimodal Data Integration

A near-term opportunity within HRM AI lies in systems capable of simultaneously integrating multiple dimensions of manometric data, pressure topography, impedance bolus transit, and clinical metadata. The current paradigm treats swallow-level classification and study-level diagnosis as sequential steps, but the full diagnostic picture emerges with peristaltic integrity, EGJ relaxation, bolus clearance, and patient-reported symptoms simultaneously. The LLM-augmented system of Popa and colleagues [12] demonstrates how generative AI can begin to contextualize raw manometric findings within broader clinical narratives, thus moving toward integrated clinical reasoning. Mascarenhas and colleagues [24] further demonstrated that training across multiple devices and centers simultaneously begins to address the platform-specific feature distributions that limit generalizability. No single manometric parameter captures the full complexity of esophageal motor dysfunction, and AI’s capacity to synthesize high-dimensional, heterogeneous pressure data across the full study may reveal diagnostically meaningful interaction patterns that current classification frameworks necessarily obscure.

### Real Time AI Assistance at the Point of Care

Real time AI that provides immediate feedback during study acquisition such as flagging probe malpositioning [13], prompting additional maneuvers when diagnostic criteria are borderline, or alerting the operator to technically suboptimal swallows, could substantially improve study quality at community laboratories where expert oversight is unavailable. The integration of LLMs into manometry interpretation [12] foreshadows a future in which AI not only classifies motility patterns but generates structured clinical reports, synthetizes findings with patient history, and suggest differential diagnosis and management. Applied across symptoms, ancillary testing, and comorbidities, this represents a meaningful step toward advancing patient-specific precision medicine in motility care along with improved clinical concordance [25]. 

### Toward Outcome-Driven AI

A key paradigm shift required is the transition from diagnosis-matched AI to outcome driven AI. Rather than optimizing models to match expert consensus diagnosis, future AI development should link manometric phenotypes to treatment response and patient reported outcomes. This mirrors the perspectives more broadly applied to healthcare AI systems, which suggests the development of a Clinical Environmental Simulator framework that would evaluate LLMs based on operational metrics and clinical outcomes [26]. Building this evidence base requires prospective cohort studies and, ultimately randomized trials to fill the current gaps in the literature.

### AI as an Equalizer: Bridging the Access Gap

AI in esophageal manometry has potential to directly address the structural access and training deficit documented in this review. Only a quarter of US fellowship programs provide formal motility training [7], and the majority of trainees complete fellowship without achieving the level of proficiency expected for independent manometry interpretation [4]. This creates a diagnostic two-tier system: expert level care at academic motility centers with NGM programs and variably qualified interpretation everywhere else.

AI’s may be advantageous through the ability to decouple interpretation quality from interpretive expertise. When an AI system trained on thousands of expert-annotated studies delivers a CCv4.0 diagnosis with 89–97% accuracy [10, 27], it effectively extends the interpretative capacity of a specialized motility unit to any laboratory equipped with manometry hardware and a network connection. The gastroenterologist in a community hospital or rural practice may gain access to a decision-support system that supplements (and in straightforward cases may substitute for) specialist oversight. This equalizing function is directly analogous to AI’s demonstrated impact in other medical fields. The global dimension of this access gap is even more pronounced in low and middle-income countries, where formal motility laboratory infrastructure is sparse and fellowship training in motility is rare or absent. AI-assisted interpretation could enable community gastroenterologists in these resource-deplete settings to perform and receive interpretive guidance on HRM studies without sending data to a remote expert or referring patients across large distances.

## Conclusion

Artificial intelligence has demonstrated compelling technical performance in HRM-based Chicago Classification automation [11, 16]. AI achieves near-expert diagnostic accuracy in controlled research settings and offers consistent, reproducible interpretation superior to human inter-rater agreement in certain tasks [6, 9]. Yet the current evidence base does not yet support clinical implementation. The complete absence of external validation [9], the lack of prospective outcome data [2], and the systematic selection bias toward high-volume expert centers [7, 12] are fundamental prerequisites for responsible clinical translation. Beyond diagnostic capabilities, AI holds transformative potential as an access and training equalizer, extending expert-level manometry interpretation to the community centers, training programs, and global settings where it is most urgently needed, and serving as a scalable tool for building the next generation of broadly competent, motility- and AI-literate practitioners.

- Gong EJ, Bang CS, Lee JJ, Baik GH. AI in Esophageal Motility Disorders: Systematic Review of High-Resolution Manometry Studies. J Med Internet Res. 2025 Nov 27;27:e85223. doi:10.2196/85223.
  - ○The field’s first dedicated systematic review of AI applied to HRM interpretation. Cited (with Fass et al.) for the central methodological critique anchoring the manuscript’s Discussion: existing AI systems are validated against expert clinical diagnosis rather than clinical outcomes, meaning the field has not escaped the “epistemological ceiling” of the CCv4.0 reference standard. As a synthesis-level source, it establishes this outcome-validation gap as field-wide rather than an isolated flaw.
- Muggleston D, O’Grady G, Varghese C, Andrews C. Artificial Intelligence in Gastrointestinal Motility Diagnostics: A Systematic Review. Neurogastroenterology Motil. 2026 Apr;38 [4]:e70310. doi:10.1111/nmo.70310.
  - ○The most recent systematic review in the bibliography, broadening scope from esophageal HRM specifically to GI motility diagnostics generally. Cited to support the claim that LLM-augmented reporting can improve “clinical concordance” in motility care, positioning it as field-level corroboration for the manuscript’s outcome-driven-AI argument.
- Mascarenhas M, Mota J, Rala Cordeiro J, Mendes F, Martins M, Cardoso P, et al. Artificial Intelligence Driven Diagnosis of Motility Patterns in High-Resolution Esophageal Manometry: A Multicentric Multidevice Study. Clin Transl Gastroenterol. 2025 Dec;16 [11]:e00941. doi:10.14309/ctg.0000000000000941.
  - ○A single (large, multicentric) primary study rather than a synthesis. Cited directly for the claim that cross-device, cross-center training begins to address the platform-specific feature-distribution shifts that limit generalizability in earlier single-center models (Kou et al., Czako et al.). The strongest direct evidence that multimodal/multicentric integration is empirically tractable now, anchoring the “Multimodal Data Integration” recommendation.

## Supplementary Information

Below is the link to the electronic supplementary material.

Supplementary Material 1 (DOCX 24.0 KB)

## Data Availability

No datasets were generated or analysed during the current study.

## Abbreviations

AI: Artificial Intelligence
ANMS: American Neurogastroenterology and Motility Society
AUROC: Area Under the Receiver Operating Characteristic Curve
BiConvLSTM: Bidirectional Convolutional Long Short-Term Memory
CC: Chicago Classification
CCv4.0: Chicago Classification version 4.0
CNN: Convolutional Neural Network
Conv3D: Three-Dimensional Convolution
DCI: Distal Contractile Integral
DES: Distal Esophageal Spasm
DL: Deep Learning
EGJ: Esophagogastric Junction
EGJOO: Esophagogastric Junction Outflow Obstruction
ESNM: European Society of Neurogastroenterology and Motility
FDA: Food and Drug Administration
FLIP: Functional Lumen Imaging Probe
FN: False Negative
FP: False Positive
HRM: High-Resolution Manometry
IEM: Ineffective Esophageal Motility
IRP: Integrated Relaxation Pressure
LLM: Large Language Model
LSTM: Long Short-Term Memory
LTHRM: Long-Term High-Resolution Manometry
ML: Machine Learning
MRS: Multiple Rapid Swallows
NGM: Neurogastroenterology and Motility
POEM: Peroral Endoscopic Myotomy
PRISMA: Preferred Reporting Items for Systematic Reviews and Meta-Analyses
SVM: Support Vector Machine
TN: True Negative
TP: True Positive
VAE: Variational Autoencoder
