# Supplementary material for: Artificial Intelligence for Diagnosis of Esophageal Manometry: A Narrative Review
Source: Curr Gastroenterol Rep. 2026 Aug 10;28(1):24. doi: 10.1007/s11894-026-01052-3 (PMC13457500; doi:10.1007/s11894-026-01052-3)
Supplement: Supplementary file 1 [file 11894_2026_1052_MOESM1_ESM.docx]

**Title:** Artificial Intelligence for Diagnosis of Esophageal Manometry: A Narrative Review

**Journal Name:** Current Gastroenterology Reports

**Author Names:**

Ernesto Robalino Gonzaga M.D., Juliana J. Madej B.S, Nitin Ahuja M.D.

Corresponding Author: Ernesto Robalino Gonzaga M.D. Division of Gastroenterology and Hepatology, Department of Medicine, Perelman School of Medicine, University of Pennsylvania, 3400 Civic Drive Boulevard, Philadelphia, Pennsylvania, USA 19104, Ernesto.Gonzaga@pennmedicine.upenn.edu

**Supplementary Information:**

Methodology:

Three databases were searched from inception to March 2026. PubMed, Embase and Cochrane. The following reproducible PubMed search terms were applied

*("esophageal manometry"[MeSH] OR "high-resolution manometry"[tiab] OR "esophageal motility"[MeSH] OR "FLIP"[tiab] OR "functional lumen imaging probe"[tiab] OR "impedance manometry"[tiab]) AND ("artificial intelligence"[MeSH] OR "machine learning"[MeSH] OR "deep learning"[tiab] OR "neural network"[tiab] OR "natural language processing"[tiab] OR "large language model"[tiab] OR "automated classification"[tiab] OR "computer-aided diagnosis"[tiab])*

Equivalent MeSH and Emtree term adaptions were applied in Embase, and Cochrane. No date restrictions were applied.
